# Cross-Section Deformation Analysis and Visualization of Shield Tunnel Based on Mobile Tunnel Monitoring System

**DOI:** 10.3390/s20041006

**Published:** 2020-02-13

**Authors:** Haili Sun, Shuang Liu, Ruofei Zhong, Liming Du

**Affiliations:** 1 Beijing Advanced Innovation Center for Imaging Theory and Technology, Key Laboratory of 3D Information Acquisition and Application, MOE, Capital Normal University, Beijing 100048, China; 2180902140@cnu.edu.cn (S.L.); limingado@163.com (L.D.); 2 College of Resource Environment and Tourism, Capital Normal University, Beijing 100048, China; 3 Academy for Multidisciplinary Studies, Capital Normal University, Beijing 100048, China

**Keywords:** point cloud data, cross-section deformation, projected images, visualization

## Abstract

With the ongoing developments in laser scanning technology, applications for describing tunnel deformation using rich point cloud data have become a significant topic of investigation. This study describes the independently developed a mobile tunnel monitoring system called the second version of Tunnel Scan developed by Capital Normal University (CNU-TS-2) for data acquisition, which has an electric system to control its forward speed and is compatible with various laser scanners such as the Faro and Leica models. A comparison with corresponding data acquired by total station data demonstrates that the data collected by CNU-TS-2 is accurate. Following data acquisition, the overall and local deformation of the tunnel is determined by denoising and 360° deformation analysis of the point cloud data. To enhance the expression of the analysis results, this study proposes an expansion of the tunnel point cloud data into a two-dimensional image via cylindrical projection, followed by an expression of the tunnel deformation through color difference to visualize the deformation. Compared with the three-dimensional modeling method of visualization, this method is easier to implement and facilitates storage. In addition, it is conducive to the performance of comprehensive analysis of problems such as water leakage in the tunnel, thereby achieving the effect of multiple uses for a single image.

## 1. Introduction

According to the Statistical Bulletin on the Development of the Transportation Industry in 2018 [1], as of the end of 2018, the operating mileage of China’s national railways reached 131,000 km. A total of 37 cities in mainland China have completed 55.19 km of urban rail lines, and tunnels and underground engineering have played an important role. At present, there are seven operating modes in China’s urban rail transit system—subway, light rail, monorail, urban express rail, modern trams, maglev transportation, and automated people mover (APM) systems, of which 4354.3 km, or 75.6%, are subway lines. With the yearly increase in the level, scale, and number of tunnels in China, the monitoring, detection, and maintenance of tunnel security status have become increasingly important. During the construction and operation of a tunnel, changes in rock and soil loads, lining support, and construction deviations will cause the tunnel to deform to a certain extent, and as the appendages on the inner wall of the tunnel age, there is a risk of dislocation and collapse. Tunnel engineering characteristically involves significant investments, high line mileage, and high concealment. Therefore, strict requirements are imposed on the quality of engineering during tunnel construction as well as on the operational maintenance [2]. Therefore, it is of considerable practical significance to study high-precision and high-efficiency methods for tunnel deformation detection and analysis.

At present, the methods of contact and non-contact measurement are used for data collection to monitor tunnel deformation. Contact measurement generally uses a total station, a level, or a convergence meter, while non-contact measurement generally uses three-dimensional (3D) laser scanner technology, digital image processing technology, or geological radar technology [3]. A total station measures several monitoring points arranged in the same cross section, and based on these monitoring points, the cross-section line of the tunnel is fitted to express the deformation of the tunnel. While deformation monitoring by the total station provides high accuracy, its efficiency as well as the density of monitoring points in the cross section are low, and thus it cannot indicate the deformation status of the inner wall of the tunnel in detail [4,5]. Terrestrial laser scanning technology is widely used for its advantages of efficiency and accuracy in data acquisition. However, multiple stations are required to be set to complete the data collection work during its application, and there must be a certain area of overlap between two adjacent stations to complete the data splicing, which generates a considerable quantity of redundant data and increases the difficulty of data processing [3].

In order to obtain the point cloud data of the inner wall of the tunnel more quickly and accurately, mobile tunnel inspection vehicles have been developed. Among them, typical vehicles are the GPR5000 tunnel scanning measurement system developed by Amberg [6] and the SiTrack: One, a mobile track scanning system [7] developed by Leica. Mobile tunnel inspection vehicles have also emerged in China, such as the Railway Mobile Measurement System (RMMS) developed at Wuhan University [8]. These systems can quickly obtain high-precision point cloud data of the tunnel section in a short time, and they collect 3D data in absolute coordinates because the systems are equipped with an inertial measurement unit (IMU) and a global positioning system (GPS) antenna. However, as deformation monitoring is based on two-dimensional (2D) sections, cross sections need to be extracted. Moreover, the cost of IMU and GPS antennas is high, limiting the popularity of the device.

In recent years, many scholars have studied algorithms for the extraction of cross sections [9,10,11,12,13,14]. Although these methods can obtain cross sections at specified positions, because of the limitations of denoising and the central axis-generating algorithm, errors inevitably occur and they affect the accuracy of the extracted cross section. Considering the advantages and disadvantages of existing equipment, this study proposes an independently developed, laser-scanner-based mobile tunnel monitoring system called CNU-TS-2. It is comprised of two odometers, a laser scanner, a gauge sensor, and a custom-made trolley, which reduces costs substantially. Because it does not require accessories such as an IMU or a GPS antenna, the system can directly obtain 2D cross-section relative position data. In addition, because an electric device is used to control the forward motion of the trolley, errors caused by the uneven speed of manual implementation are effectively avoided. The accuracy of the system was validated by comparing the data collected by the system with the data obtained by a total station. 

With the increasing popularization of 3D laser scanning technology in tunnel detection, algorithm research in deformation analysis and visualization has also increased. At present, deformation analysis primarily involves four methods: using the least square method to fit a circle, fitting an ellipse by segments or by the whole, and fitting various curves. Although the cross section of the tunnel is designed to be circular, it will be slightly deformed after its completion owing to environmental and construction influences. Therefore, Lindenbergh [15] proposed a method to fit a circle for deformation monitoring based on laser point cloud data. Compared with the single-point measurement method, this method improves the analysis accuracy to a certain extent, but the deformed section is no longer an ellipse. D. Delaloye [16], Walton [17], Li Cheng [18], Li Jian [19], and Xie Xiongyao [20] proposed that the overall fitting of the ellipse for deformation analysis achieved good results. While the method of piecewise fitting of an ellipse proposed by Li Jiaping [21] and Zhang Lishuo [22] is more rigorous than the overall fitting, the need to mark the position of each segment significantly increases the workload, and thus it cannot be applied to engineering. Liu Guanlan [23] used curve fitting to deform the tunnel section, but this method is highly limited by the number of appendages in the tunnel and the effect of denoising. Therefore, in the present study, the currently widely-used global fitting ellipse was chosen for deformation analysis. The visualization of tunnel deformation can be divided into two forms: 2D and 3D. Zhang Fan [24] and Wang Lingwen [25] realized 2D visualization by showing the deformation of a single cross section; 3D visualization was mainly realized through modeling [20,26].

In this study, a new 2D method for visualizing deformation is proposed. This method uses data from the entire tunnel to depict deformation. Here, a shield tunnel is used as the research object, the section point cloud data are used as the basis, and a single section is used as the analysis unit for the deformation analysis by the algorithm for the least-squares fitting of an ellipse. Then, by applying a cylindrical projection method, the deformation values are mapped to a grayscale image using different colors to achieve visualization. This visualization method enables the evaluation of tunnel safety by combining tunnel defects, and displaying the deformation data from the tunnel in an engineering-friendly manner. It also provides data that offer a basis for detecting the tunnel safety status.

## 2. The Mobile Tunnel Monitoring System

### 2.1. Integrated System Hardware 

**Figure 1:**
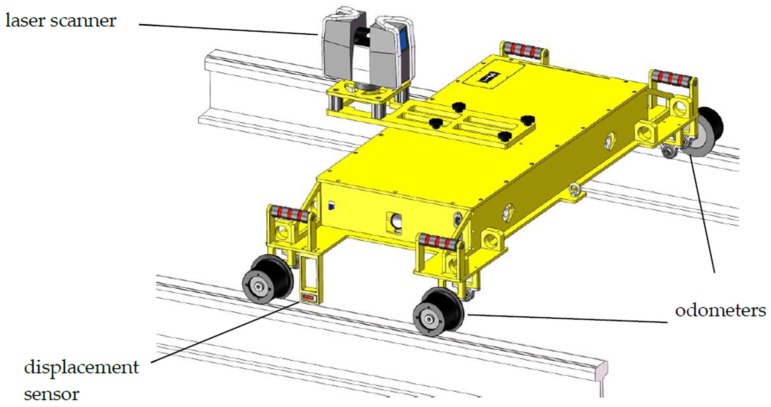
Mobile tunnel monitoring system—CNU-TS-2.

This study presents an independently developed a mobile tunnel monitoring system called CNU-TS-2. The system (Figure 1) consists of a laser scanner, two odometers, a displacement sensor, an electric system, an industrial notebook, and a custom-made trolley. As shown in Figure 1, it is developed by considering that mobile laser scanning systems are highly efficient and have the ability to quickly acquire a large quantity of data, while simultaneously taking advantage of the characteristics, functions, applicability, and practicality of various sensors. The size of the trolley is about 60 cm × 155 cm × 40 cm, and its forward speed is 0.45–5.4 km/h.

This system can utilize the Faro, Leica, and Z + F laser scanners as the primary measurement equipment. The parameters for each scanner are shown in Table 1. During the mobile measurement process, the scanner operates in a 2D spiral pattern, which can meet the needs of various scenarios such as tunnel tracks. The system also uses a stepper motor as an odometer to perform mileage calculations. The distance can be calculated from the wheel radius and the number of rotations made by the wheel, which is important for the mileage of the trolley. In order to improve the accuracy of the mileage data, an odometer is installed on the left and right sides of the rear wheel of the trolley. In addition, the system uses the Banner LT3 series laser displacement sensor for gauge measurement. The measurement resolution of the system can reach 0.005 mm and the gauge data obtained can be used for the later detection of the tunnel headroom.

### 2.2. System Software Implementation

**Figure 2:**
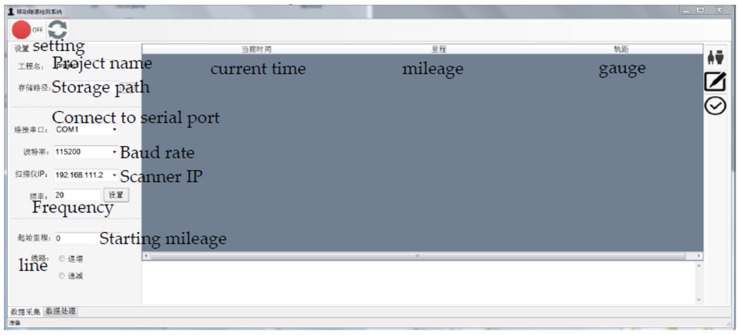
Data acquisition software.

Data acquisition software for controlling hardware devices and data processing software for data analysis were developed under the Visual Studio 2013 C ++ and Qt platforms, enabling a variety of sensors to be controlled and the data collection task to be completed in order to obtain usable point cloud data and meet the relevant requirements for the processing and analysis of the obtained data. The interface of the data acquisition software is shown in Figure 2. The software includes three main modules: the setting module, data acquisition module, and data pre-processing module. In the actual measurement process, time synchronization for the multiple sensors is achieved by setting the relevant parameters for each sensor, such as IP address, storage path, and file name. On this basis, data collection and storage are performed. Then, the point data collected by the laser scanner in spiral mode (whose *y* coordinate is zero) are expanded along the *y*-axis by the mileage data collected by the odometer based on the timestamp. Finally, the 3D cross-section point cloud data are obtained in LAS format (A standard Light Detection And Ranging (LIDAR) data format which was released by the LIDAR committee of the American Society for Photogrammetry and Remote Sensing (ASPRS) in 2003.) for later analysis. 

**Figure 3:**
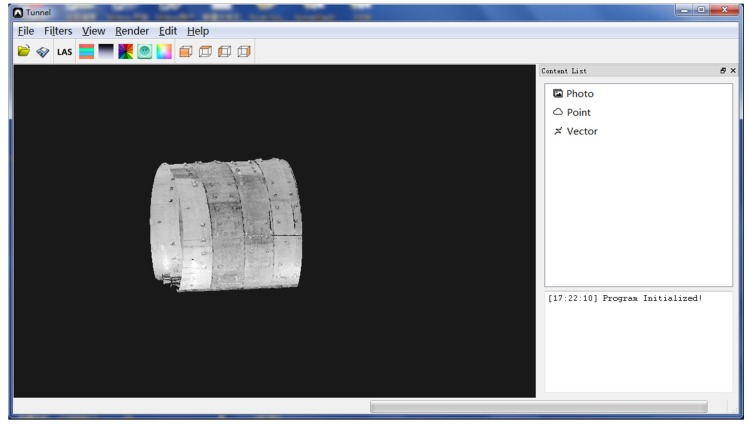
Data processing software.

After obtaining the point cloud data using the data acquisition software, all the data or a single cross section at the specified location can be processed by the software (Figure 3), which can execute operations such as data display, denoising, mileage correction, projected images generation, cross-section analysis, contrast analysis, and limit detection to meet daily testing needs. The data processing software interface is shown in Figure 3.

## 3. Methods

**Figure 4:**
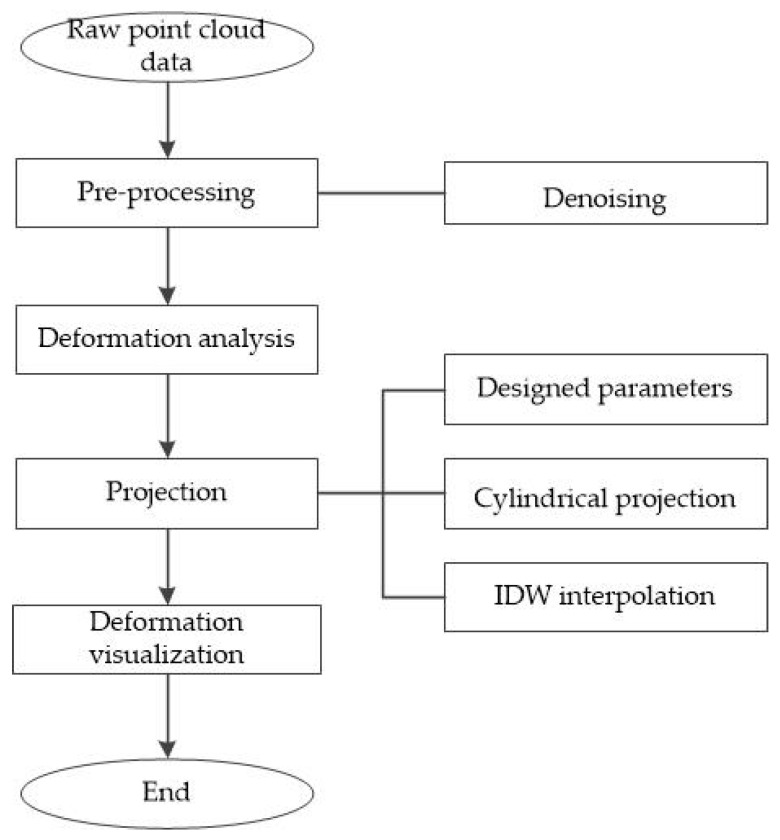
Flow chart of the proposed method. IDW, inverse distance weighting.

This study first analyzes the deformation of the cross-section point cloud data collected by the CNU-TS-2 mobile tunnel monitoring system to obtain the deformation values from the measured data and the design data of the tunnel, and then compares this deformation with the corresponding total station data to validate the system’s accuracy. Subsequently, the tunnel point cloud data are expanded using the cylindrical projection method, and the grayscale values of the point cloud data are interpolated using the inverse distance weighting (IDW) method to generate a grayscale image. Finally, the deformation value is used as a constraint condition, and the deformation value is represented by a particular color gradient in the grayscale image through a linear transformation to achieve the visualization of the deformation value. The overall work flow for the method is shown in Figure 4.

### 3.1. Deformation Analysis Method

The convergent diameter of a shield tunnel is an important indicator in the measurement of the lateral deformation of the tunnel. Thus, the proposed method analyzes the deformation of the tunnel by calculating the convergent diameter of each ring of the tunnel using the measured point cloud. At the same time, in order to more comprehensively reflect the overall deformation characteristics of the tunnel and provide more powerful data support for the analysis of the causes of deformation in a later stage, the proposed method includes a technique for analyzing the overall deformation of the tunnel.

#### 3.1.1. Denoising

In the actual scanning operation, owing to the obstruction of obstacles or the limitation of the scanning angle of the tunnel space, a portion of the point cloud data that does not belong to the tunnel wall—that is, noise points—appears, owing to the obstruction by obstacles or limitations on the tunnel space scanning angle. As these noise points seriously affect the tunnel deformation analysis in the later stage, they need to be eliminated. At the same time, the deformed tunnel cross section can be considered as an ellipse close to a standard circle [17,27]. Therefore, the proposed method uses the technique of iteratively fitting an ellipse to remove cross-section noise points. The parameter estimation during ellipse fitting uses the random sample consensus (RANSAC) algorithm [28]. The steps are as follows:

First, the algorithm for least-squares fitting of an ellipse is used to fit the noisy cross-section point cloud, so that the initial parameters of the ellipse (including the coefficients of the ellipse equation, the coordinates of the center of the circle, and the long and short semi-axes) can be calculated. The principle is shown in Appendix A.

The shortest distance $d_{i}$ from the section point to the ellipse is solved, forming the distance point set $d\left\{d_{1}{,d}_{2}{,\ldots ,d}_{m}\right\}$. Next, the mean $d_{mean}$ and standard deviation σ of the point set are calculated:

(1) $$d_{mean}=\frac{\sum d}{m},$$

(2) $$\sigma =\sqrt{\frac{1}{n}\sum_{i=1}^{n}{{(d}_{i}{-d}_{mean})}^{2}}.$$

It is stipulated that, when $|d_{i}{-d}_{mean}|>2\sigma$, it is a noise point. After a series of continuous iterations, the points not on the tunnel wall are finally removed.

#### 3.1.2. Calculation of Deformation 

**Figure 5:**
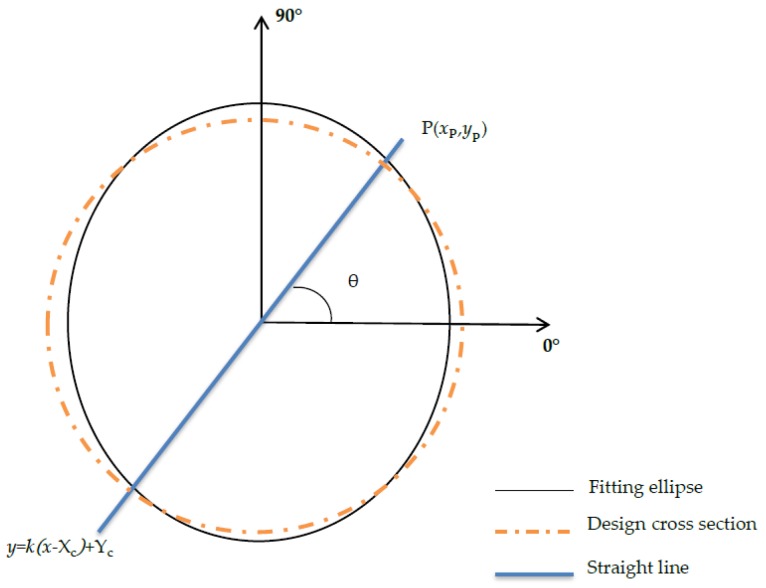
Calculation of deformation value for cross-section points at each angle.

In the proposed method, the deformation analysis for each cross section is performed by fitting an ellipse (Figure 5). The method can use the result of the denoising fitting directly to reduce the amount of calculation and improve the operation efficiency. Compared with the method of calculating the deformation by fitting circles, this technique is more suited to the characteristics of tunnel deformation and is more reasonable. When the deformation values of the section at various angles are calculated, the center of the section-fitting ellipse is taken as the origin, and the circle’s center is taken as the starting point. On this basis, the angle between each section point and the *x* axis in the section’s local coordinate system is calculated. First, at 0.5° or 1° intervals, a straight line is made through the origin in the direction from 0° to 360°.

(3) $${y=k(x-X}_{c}{)+Y}_{c},$$

where ${(X}_{c}{,Y}_{c})$ are the coordinates of the center of the fitted ellipse, and k=tanθ is the slope of the straight line, in which θ is the angle between the straight line and the positive *x*-axis in a counterclockwise direction. Then, by applying the ellipse equation (Equation (A1)), the intersection point ${P(x}_{p}{,y}_{p})$ between the straight line and the fitted ellipse is found, and next, the distance from the point in each direction on the ellipse to the center of the ellipse is obtained according to the following:

(4) $$d=\sqrt{{{(x}_{p}{-X}_{c})}^{2}{+(y}_{p}{-Y}_{c}{)}^{2}}.$$

where d is then compared with the design radius to obtain the deformation value in each direction. A deformation value whose distance value is greater than the theoretical radius of the segment is deemed positive; otherwise, it is deemed negative. On the basis of this criterion, the deformation value is identified as positive or negative, and the result is assigned to the point cloud data.

### 3.2. Projected Images Generation Method

As each cross section of the single-circle shield tunnel data can be fitted to an ellipse whose shape is close to that of a standard circle, we can abstract the point cloud data of the single-circle shield tunnel in rings as a set of standard cylinders [5]. The side of each cylinder is developed to a plane according to the principle of mathematical projection. The process is divided into the following steps.

#### 3.2.1. Calculation of Fitted Circle Parameters

During circle fitting, the RANSAC algorithm is used for parameter estimation. First, through random sampling, three points are randomly selected from the point cloud of the section and brought into the following equation.

(5) $${{(x-x}_{c})}^{2}{+(y-y}_{c}{)}^{2}{=r}^{2},$$

where ${(x}_{c}{,y}_{c})$ are the coordinates of the center of the circle, (x,y) are the coordinates of the sampling point, and *r* is the radius of the fitted circle. Then, the initial parameters of the fitted circle (the coordinates of the center of the circle ${(x}_{0}{,y}_{0})$ and the radius $r_{0}$) are obtained, and the parameters of the best-fitted circle are obtained through dozens or even hundreds of iterations. Considering that the tunnel segment is made of prefabricated reinforced concrete, the deformation in the same ring has little effect on the fitted circle. In order to improve the calculation efficiency, only the middle position of each ring is used to calculate the circle parameters.

#### 3.2.2. Projection of Tunnel Point Clouds

**Figure 6:**
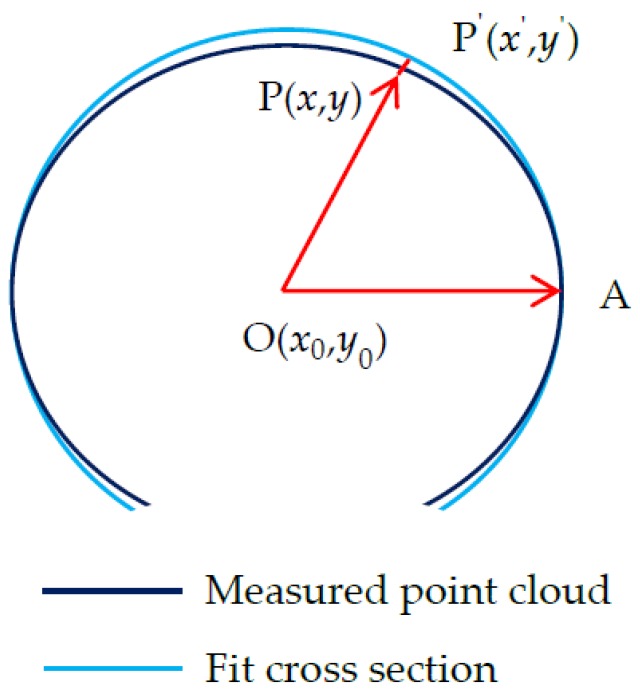
Cross-section point cloud projection.

The cross-section point cloud is projected onto the fitted circle section, as shown in Figure 6. $\;\vec{\mathrm{OA}}=(1,0)$ is the *x*-axis positive unit vector, P=(x,y) is a point in the point cloud, and P′=(x′,y′) is the projection point of P; thus,$\;\vec{\mathrm{OP}}{=(x-x}_{0}{,y-y}_{0})$. ∠POA is positive when it rotates counterclockwise around the *x* axis. Therefore, P′=(x′,y′) for the projection can be obtained according to the following equations:

(6) $$\{\begin{array}{c} x\prime =R\cdot cos\theta \\ y\prime =R\cdot sin\theta , \end{array}$$

(7) $$\theta =\;∠\mathrm{POA}\;={\;\cos}^{-1}\frac{\vec{\mathrm{OP}}\cdot \vec{\mathrm{OA}}}{\left|\vec{\mathrm{OP}}\right|\cdot \left|\vec{\mathrm{OA}}\right|}.$$

**Figure 7:**
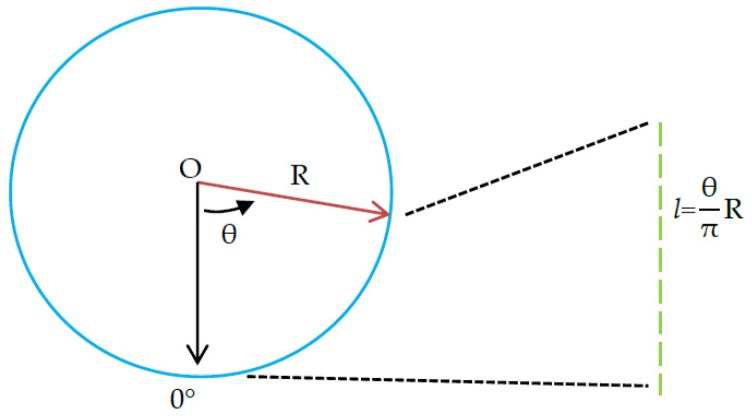
Diagram showing expansion of cross-section point cloud.

**Figure 8:**
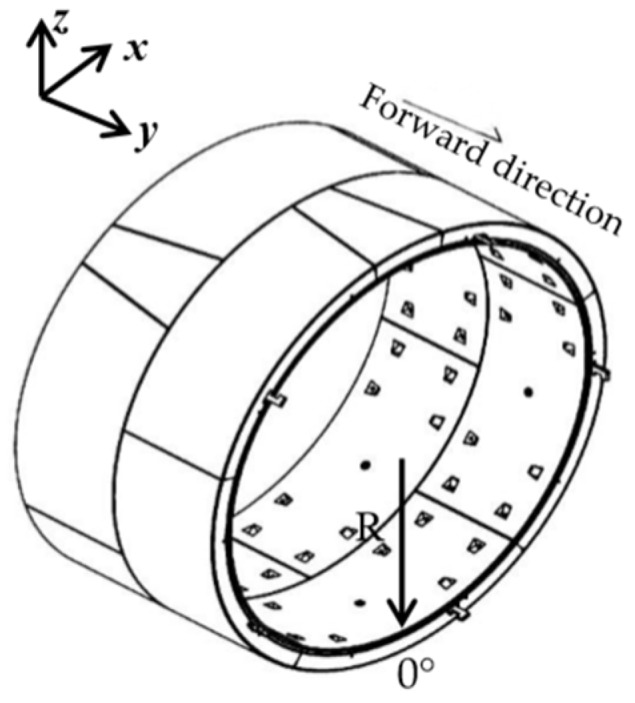
Diagram of tunnel segment structure.

**Figure 9:**
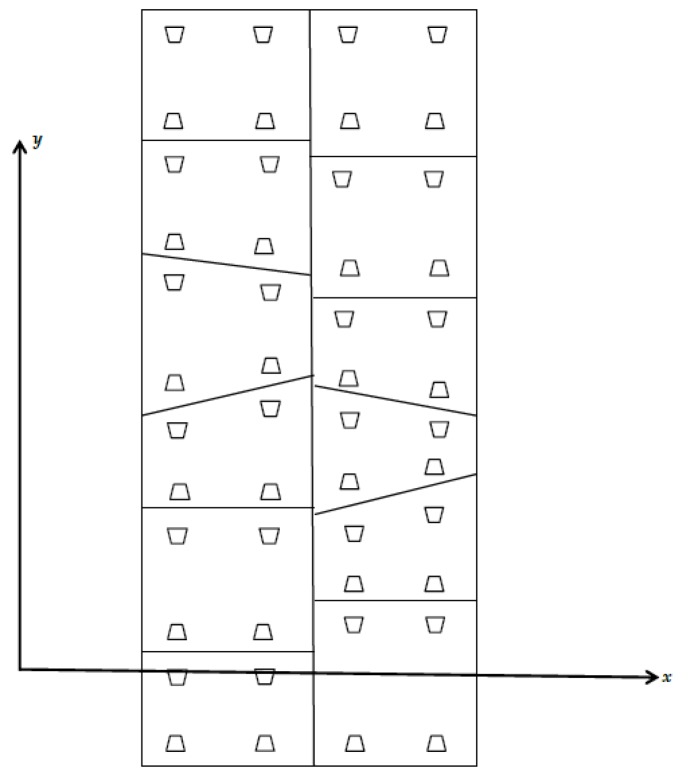
Diagram of cylindrical projection.

After the projection, taken perpendicular to the circle’s center and pointing downwards as the starting direction, the counterclockwise direction is positive. The denoising tunnel point cloud is expanded from section to section. Finally, two-dimensional point cloud data with the forward direction as the *x*-axis and the cross-section arc length *l* as the *y*-axis are obtained, where arc length $l=\frac{\theta }{\pi }R$, in which θ is the vertical angle and R is the radius of the fitted circle. See Figure 7, Figure 8 and Figure 9 for explanatory diagrams.

#### 3.2.3. Generation of Images

First, the coordinates of the long side, short side, and minimum corner point of the two-dimensional point cloud are found according to the smallest circumscribed rectangle; the minimum corner point is then used as the image origin. Then, according to the actual size represented by one pixel, the numbers of pixels in the length and width of the generated image are determined. Because point cloud data are discrete, in order to ensure that each pixel in the image has a corresponding intensity value, inverse distance weighting (IDW) is used in this paper. Using the center point of each pixel as the center and two pixels as the radius size, the intensity for the two-dimensional point cloud data in the range is weighted to obtain the intensity value of the current pixel point, using the formulas given as Equations (8)–(11). Finally, the image is rotated and output according to the angle between the image and the *x-*axis.

The general formula of the inverse distance weighting algorithm is

(8) $$\hat{Z}(S)=\sum_{i=1}^{n}W_{i}Z(S_{i})\;,$$

where $\hat{Z}(S)$ is the predicted value at the pixel center point S(*x,y*), *n* is the number of discrete points within a certain range from the pixel center point during the prediction calculation process, $w_{i}$ is the weight of each discrete point used in the prediction calculation process, and ${Z(S}_{i})$ is the intensity value at the discrete point $S_{i}{(x}_{i}{,y}_{i})$. The weight formula is

(9) $$w_{i}=\frac{h_{i}^{-p}}{\sum_{i=1}^{n}h_{i}^{-p}}\;,$$

(10) $$\sum_{i=1}^{n}w_{i}=1.$$

where *p* is an arbitrary positive real number; and $h_{i}$ is the distance from the discrete point to the pixel center point, given by

(11) $$h_{i}=\sqrt{{{(x-x}_{i})}^{2}{+(y-y}_{i}{)}^{2}},$$

where (*x*,*y*) are the coordinates of the pixel center point, and ($x_{i}{,y}_{i}$) are the coordinates of the discrete point.

### 3.3. Deformation Visualization

In order to intuitively reflect the deformation of the inner wall of the tunnel on the projected image, the proposed method will visualize the deformation values of the inner wall of the tunnel in three colors: red, green, and blue. Simultaneously, in order to improve the visualization effect, the deformation values will be represented by particular color gradients. This technique is implemented as follows.

First, when the point cloud data are rasterized, the method for interpolating the deformation value is the same as that used to generate the grayscale image (described in Section 3.2). Then, the grayscale value is mapped into the corresponding three channels of red, green, and blue according to the size of the deformation value, and the generated image will display different colors according to the deformation values. However, in order to reflect differences in the amounts of deformation for each case, the grayscale values in each channel are linearly transformed so that the colors in the generated image are displayed with a gradient, thereby improving the visualization effect.

## 4. Method Validation

### 4.1. Data Sources

The validation data consist of two data sets. One is the Chengdu data set, used to verify the accuracy of the mobile tunnel monitoring system, and the other is the Tianjin data set, used to verify the deformation analysis and its visualization method.

#### 4.1.1. Chengdu Data Set

**Figure 10:**
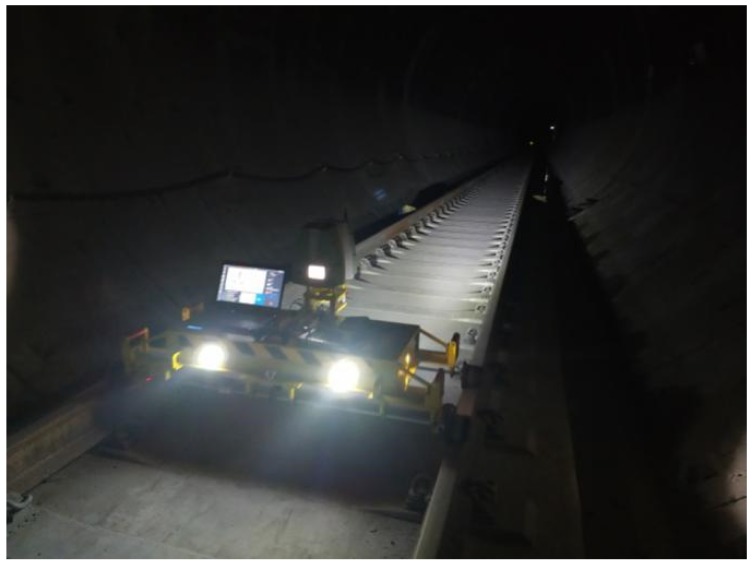
Photo of the scene.

**Figure 11:**
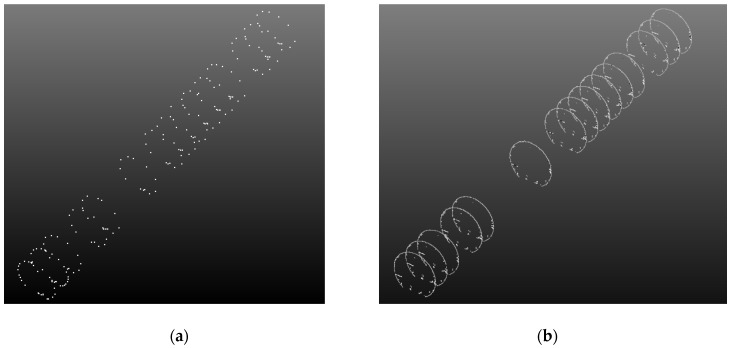
Cross-section data: (**a**) total station data; (**b**) corresponding cross-section point cloud data.

For this test, a metro tunnel in Chengdu (Sichuan, China) was measured. The tunnel (Figure 10) is a circular shield structure with a designed inner diameter of 5.4 m and a ring width of 1.5 m. The segments of the tunnel, which are made of concrete, are spliced using a staggered method, and the measured length is 100 m. The system is equipped with a Faro Focus 3D 120 to measure at a speed of 1.8 km/h. During the test, a total station was used to synchronously acquire section data in the survey area. Owing to time constraints, some cross-section measurements were abandoned. Finally, fifteen cross sections were measured at intervals of two to three meters, as shown in Figure 11a. The corresponding cross-section data measured by the proposed mobile tunnel monitoring system are shown in Figure 11b.

#### 4.1.2. Tianjin Data Set

**Figure 12:**
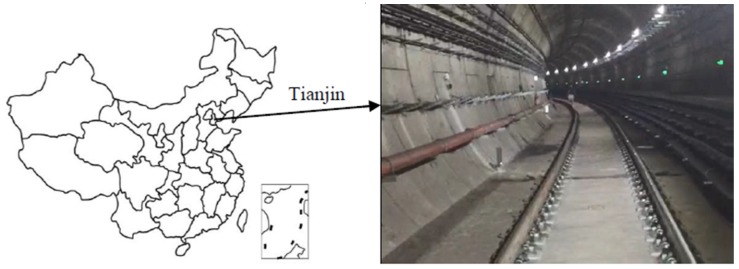
Location diagram map and photo of test location.

This test was performed in a circular shield tunnel of the Tianjin Metro, the location of which is shown in Figure 12. This section of the tunnel began operation in October 2018. Its tunnel lining was spliced using a staggered joint method. Its design inner diameter is 5.5 m and the ring width is 1.5 m. In addition, there are cables, pipes, power equipment, escape platforms, lighting equipment, drainage ditches, and other equipment and installations in the tunnel.

**Figure 13:**
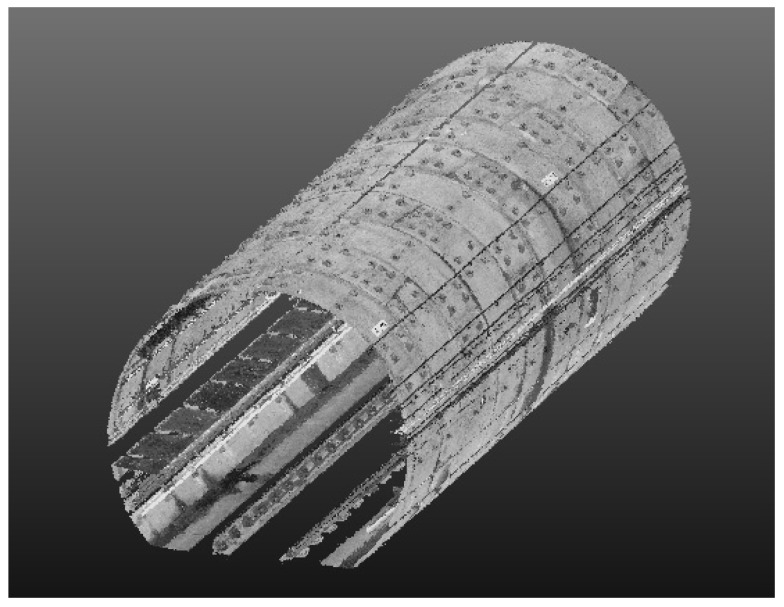
Part of Tianjin metro tunnel point cloud data.

The range of operation is K31,346 to K31,557.5, with a total of 141 rings and a total length of approximately 211.5 m. CNU-TS-2 equipped with Faro Focus 3D 120 was used to take round-trip measurements at a speed of 0.9 km/h. After the data collection was complete, the system software was used to perform data fusion to obtain the tunnel point cloud data in relative coordinates. The data were in LAS format, including *x*, *y*, *z*, and intensity information. The point cloud data are depicted in Figure 13.

### 4.2. Validation of Accuracy of CNU-TS-2 by Total Station

**Figure 14:**
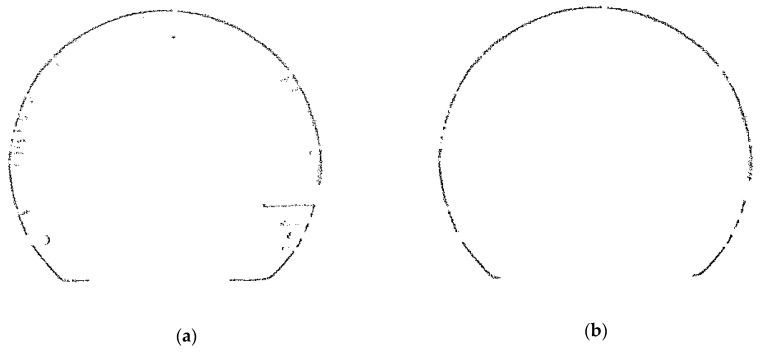
Single-section point cloud data:(**a**) before denoising; (**b**) after denoising.

Because there are many auxiliary facilities in the tunnel, the section data needed to be denoised before deformation analysis. The denoising result of a single cross section is shown in Figure 14.

First, the ellipse was fitted to the two sets of cross-section data. Next, the coordinates of the 0° and 180° positions were obtained according to the elliptic equation obtained after the fitting, and then the convergence diameter was obtained. The accuracy of the tunnel movement monitoring system was validated by examining the deviation of its results from the convergence diameters as measured by the total station for the same location. The analysis results are shown in Table 2. It can be seen that the average absolute deviation from the convergence diameters obtained by two devices is 1.32 mm. According to the Technical Specification for Urban Rail Transit Engineering Monitoring [29], the accuracy requirement for metro tunnel deformation detection is ±3 mm. Thus, the CNU-TS-2 mobile tunnel monitoring system can fully meet the accuracy requirements for metro tunnel detection.

### 4.3. Validation of Deformation Analysis and Visualization Method 

#### 4.3.1. Validation of Deformation Analysis Repeatability 

**Figure 15:**
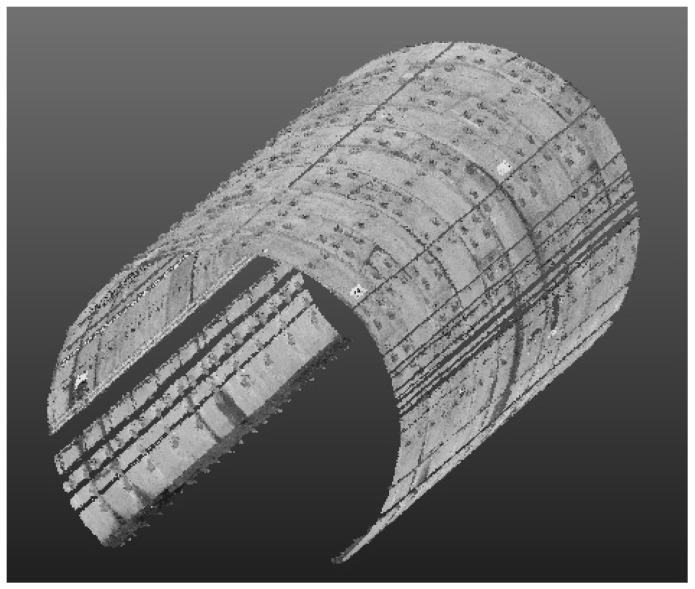
Denoising diagram for part data set.

The repeatability of the CNU-TS-2 mobile tunnel monitoring system was validated by comparing the local or overall deformation analysis results from the round-trip measurements of the tunnel section in the experimental area. The entire experimental area had a total of 141 rings of data, and each ring contained 200–300 sections, so the quantity of data was quite large. Therefore, in the two round-trip measurements, only the horizontal convergence diameter of the middle mileage section of each ring was taken to represent the repeatability of the method in a single direction. Before performing the deformation analysis, the data needed to be denoised. The effect of denoising is shown in Figure 15.

**Figure 16:**
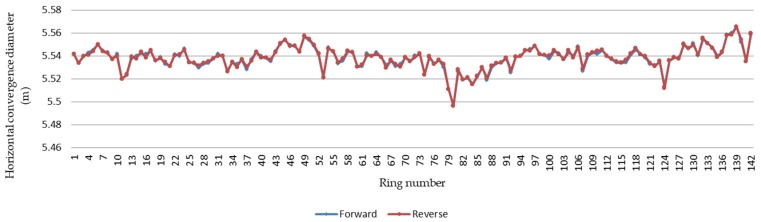
Repeatability of convergence diameter results by ring.

**Figure 17:**
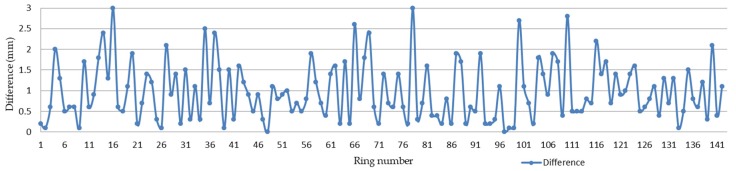
Graph of difference between convergence diameter results by ring.

By comparing the repeatability of the horizontal convergence diameters of the round-trip measurements, it was found that the absolute deviation between the measurements was between 0 mm and 3 mm, and the average value was 1 mm. Rings for which the difference was less than 1 mm account for 57.75% of the total data, those with a difference between 1 mm and 2 mm account for 33.80%, and those with a difference greater than 2 mm account for 8.45%. The repeatability of the convergent diameter results for each ring is shown in Figure 16, and the difference in the convergent diameter results for each ring is shown in Figure 17.

**Figure 18:**
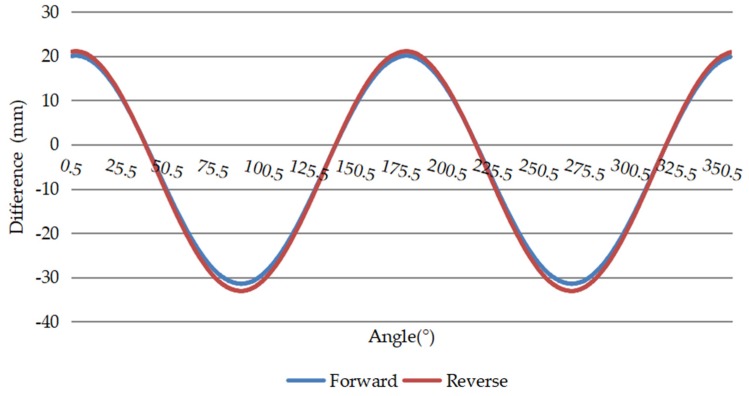
Graph displaying repeatability of single-section deformation values.

The repeatability of the deformation analysis for a cross section at the same position is represented by a comparison of two measurements of the deformation value for a single cross section at 360° from various angles, as shown in Figure 18.

#### 4.3.2. Projected Images Quality Evaluation

**Figure 19:**
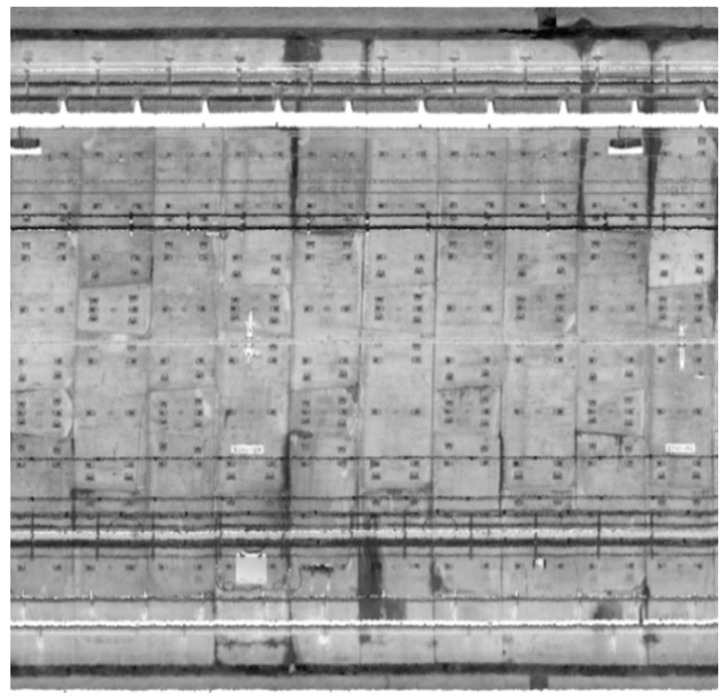
Grayscale projected image.

The projected images generated by the proposed method represent the actual distance of each pixel at 5 mm × 5 mm. Compared with direct acquisition by a camera, this method is advantageous because there is no need for image correction, stitching, or a light source. The effect of grayscale imaging is shown in Figure 19. 

There are two main aspects of image quality: the fidelity of the image and the intelligibility of the image. Image quality is directly affected by the optical performance of the imaging equipment, image contrast, instrument noise, and other factors. Quality evaluation can provide monitoring means for image acquisition, processing, and other steps. As a way to reasonably evaluate each link in the chain of image processing, image quality evaluation has become a basic technology of image information engineering.

1. Subjective Evaluation

The subjective quality scoring method [30] is the subjective method most typically used for evaluating image quality. It determines image quality by normalizing observers’ scores. Subjective quality scoring methods can be divided into two types: absolute evaluations and relative evaluations. Absolute evaluations grade the image directly according to visual perception. Table 3 lists the two five-level absolute scales specified internationally; that is, the quality scale and the obstacle scale.

**Figure 20:**
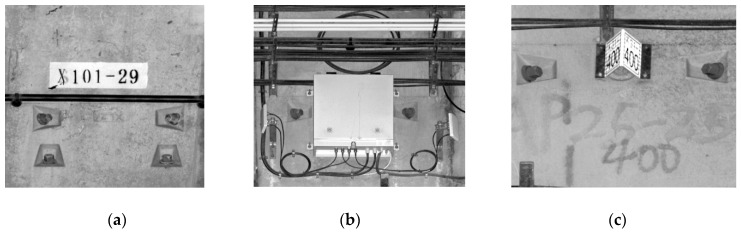
Enlarged views of grayscale projected images: (**a**) sign on the inner ring of the tunnel; (**b**) communication equipment; (**c**) a mileage plate.

By directly observing Figure 19 and the enlarged views shown in Figure 20, it can be seen that the grayscale image generated by interpolating the grayscale values of the point cloud is similar in quality to a camera image. The image does not show any deterioration in quality, clearly distinguishes between the appendages and distressed areas in the tunnel, and can meet the requirements of daily inspection.

2. Objective Evaluation

In the objective evaluation of image quality, a mathematical model is established based on the subjective visual system of the human eye, and the image quality is calculated using a specific formula [30]. In contrast with subjective evaluation, objective evaluation allows batch processing, and the results are more reproducible, with no deviations because of contrived reasons. In the proposed method, three indicators are selected—the mean, standard deviation, and average gradient—to evaluate the quality of the image.

(1) Mean

The mean is the average value of the image pixels. It represents the average brightness of the image. The higher the average brightness, the better the image quality.

(12) $$u=\frac{1}{MN}\sum_{i=1}^{M}\sum_{j=1}^{n}F(i,j).$$

(2) Standard Deviation

Standard deviation refers to the degree of dispersion of the grayscale value of an image pixel relative to the mean. A higher standard deviation means that the grayscale levels in the image are more dispersed, and the image quality is better.

(13) $$std=\sqrt{\frac{1}{MN-1}\sum_{i=1}^{M}\sum_{j=1}^{n}{(F\left(i,j\right)-u)}^{2}}.$$

(3) Average Gradient

The average gradient expresses the contrast of details and texture transformation in the image, and to a certain extent, it can represent the clarity of the image. Generally, the higher the average gradient, the more changeable, richer, and clearer the image.

(14) $$\nabla G=\frac{1}{MN}\sum_{i=1}^{M}\sum_{j=1}^{n}\sqrt{\Delta xF{\left(i,j\right)}^{2}+\Delta yF{\left(i,j\right)}^{2}},$$

where

$$\{\begin{array}{c} \Delta x=\frac{\left[F\left(i+1,j\right)-\;F\left(i-1,j\right)\right]}{2}\\ \Delta y=\frac{\left[F\left(i,j+1\right)-\;F\left(i,j-1\right)\right]}{2} \end{array}.$$

MATLAB R2015a was used to calculate the above values for the image in Figure 19; the mean was 156.2106, the standard deviation was 44.8846, and the average gradient was 0.0158. 

Using a combination of subjective and objective evaluation methods, it can be concluded that the projected image of the inner wall of the tunnel obtained by the cylindrical projection method described in this paper can clearly identify various auxiliary facilities and diseases in the tunnel, indicating that the image quality is good.

#### 4.3.3. Analysis of Deformation Visualization 

**Figure 21:**
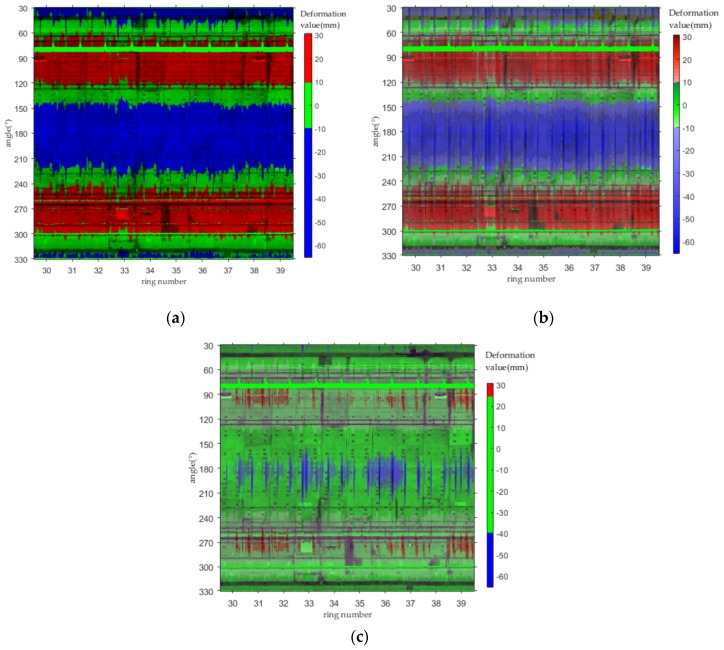
Deformation visualization for projected images: (**a**) deformation map using three primary colors (red, green and blue); (**b**) deformation map using color gradients; (**c**) deformation map using three primary colors showing deformation values outside (−40,25).

Deformation analysis was performed on the point cloud data corresponding to Figure 19 using the method described in this article. The range of the deformation value was (−65.05,30.59), and the unit was mm. The deformation value was interpolated for each pixel by inverse distance weighting (IDW). In the deformation visualization, (−10,10) is displayed in green, which means that the deformation in this range is normal. The part with deformations less than −10 mm is represented as blue, and the remaining part, with deformations greater than 10 mm, is represented as red. The resulting display is shown in Figure 21a. In order to cause the image to display as much information as possible, each color (red, green, and blue) is divided into gradients. The darker the color, the higher the deformation value it represents; conversely, the lighter the color, the lower the deformation value. The resulting display is shown in Figure 21b. From Figure 21b, it can be seen that the blue part is generally lighter in color and the color gradient does not change very much, indicating that the differences between the deformation values less than –10 mm are small. In the same way, the color of the red part is uniform and is not dark, which indicates that the differences between the deformation values larger than 10 mm are small. Finally, Figure 21c shows the effect of changing the range of the green area to (−40,25) and demonstrates that the deformation values in this data set are concentrated mainly within (−40,25).

## 5. Results

This study proposes a mobile tunnel monitoring system, called CNU-TS-2. The system consists of a laser scanner, two odometers, a displacement sensor, an electric system, an industrial notebook, and a custom-made trolley. Its advancement is controlled by an electric system, and its travel speed is 0.45–5.4 km/h. It can also be compatible with a variety of scanners to meet the accuracy requirements of different projects. This article described the accuracy of data collected by the device and visualization of deformation analysis in two tests. The first was the Chengdu test. After denoising, the convergent diameter values of the 15 sections collected by the total station were compared with the corresponding positions collected by this system. Its average absolute deviation was 1.32 mm, which is in good agreement industry standards; thus, the accuracy of the data collected by the system was verified. The second was the Tianjin test. After denoising, the least square method for fitting ellipses was used to analyze the deformation of all the data. It can be seen from Figure 18 that the differences between the reverse and forward of the single cross section were small. Then, comparing the difference in the convergent diameter of the middle reverse and forward cross sections of the 141 loop data of the entire measurement area, it can be concluded that the method is feasible. Figure 17 shows that the difference between the round-trip convergence diameter of the middle section of the 141 loop data of the entire measurement area is within a reasonable range. Therefore, it can be concluded that this deformation analysis method is feasible. Finally, using the deformation value as a constraint, the point cloud is expanded by cylindrical projection, and a color image is obtained by IDW interpolation to realize the visualization of the deformation.

## 6. Discussion and Conclusions

In this study, it was experimentally demonstrated that the CNU-TS-2 mobile tunnel monitoring system can achieve high accuracy and repeatability. Unlike existing trolleys [31,32,33], the forward movement of this trolley is controlled through the system’s electric devices, and thus the impact of an uneven walking speed on the generation of point cloud data is substantially reduced. Additionally, the system can be equipped with a variety of laser scanners to meet the data requirements of different projects. However, because of working limitations, the system is not equipped with an IMU or a GPS antenna, and thus the point cloud data obtained are only positioned relative to the tunnel. Although the data are strictly corrected by the odometers, the positioning accuracy requires further improvement. Nevertheless, in practical engineering applications, point cloud data with relative locations can effectively be used for daily monitoring of tunnel deformation, and it is easier to perform cross section-based data analysis using the data generated by this system. Owing to the limitations of the working environment, the system cannot be loaded with inertial navigation components to obtain the attitude information of the trolley. In the future, consideration will be given to adding an inclinometer to obtain the attitude of the trolley and integrate it into the point cloud data. Another limitation is that this trolley is only suitable for use on 1435 millimeter railways or subways. In order to expand its applicability, its body design is expected to be improved subsequently to make it suitable for use on tracks of other widths.

In addition to evaluating the feasibility of the data obtained by the mobile tunnel monitoring system, this study also denoised the tunnel data by fitting ellipses using random sample consensus. The fitting parameters—such as the ellipse center and the long and short semi-axes—can also be used for deformation analysis, which reduces the amount of calculation required. Following the deformation analysis, the analysis results are enriched by mapping the deformation values to the projected image of the inner wall of the tunnel and distinguishing them using different colors. Compared with existing methods for displaying deformation analysis results based on 3D models [18,19], this method better facilitates the storing and viewing of results. More importantly, it facilitates comprehensive analysis of problems such as water leakage and cracks in the image, thereby better achieving the effect of multiple uses of a single image. Nevertheless, several improvements remain to be made to the algorithm. For example, the algorithm is currently only suitable for shielded circular tunnels; however, because of the variety of excavation methods, there are also tunnels shaped as horseshoes, rectangles, and arches. In order to improve the universality of the system, it is necessary to further study deformation analysis methods for use with other tunnel shapes. Second, in order to reflect the true deformation for each section (and to allow further analysis of the deformation characteristics of sections in the same ring), the algorithm calculates the deformation for all sections, and as each ring of data has three or four hundred sections, this leads to a large amount of data calculation. In future versions of the method, the deformation characteristics of cross sections within the same ring can be further analyzed to sample the data in the ring to reduce the amount of calculation required, thereby improving the calculation efficiency.

## Figures and Tables

**Table 1:** Scanner-related parameters.

Name	Ranging Accuracy	Scan Speed (points/s)	Angle Measurement Accuracy	Vertical Field of View
Faro Focus 3D 120	±2 mm	97,600	0.009°/0.009°	300°
Leica ScanStation P30/P40	±1.2 mm	1,000,000	0.002°/0.002°	290°
Z + F IMAGER 5016	±1 mm	1,100,000	0.004°/0.004°	320°

**Table 2:** Comparison of convergence diameters as measured by total station and by CNU-TS-2.

Ring Number	Diameter: Total Station (m)	Diameter: CNU-TS-2 (m)	Absolute Deviation (mm)
1	5.4133	5.4153	2.0
2	5.4139	5.4131	0.8
3	5.4158	5.4134	2.4
4	5.4105	5.4096	0.9
5	5.4144	5.4154	1.0
6	5.4162	5.4147	1.5
7	5.4141	5.4126	1.5
8	5.4138	5.4152	1.4
9	5.4139	5.4133	0.6
10	5.4136	5.4157	2.1
11	5.4134	5.4155	2.1
12	5.4084	5.4093	0.9
13	5.4137	5.4118	1.9
14	5.4155	5.4156	0.1
15	5.4139	5.4133	0.6

**Table 3:** Absolute scales for subjective evaluations of image quality.

Quality Scale		Obstacle Scale	
5 points	No image degradation is noticed	5	Very good
4 points	Can see changes in image quality without hindering viewing	4	Good
3 points	It is clear that the image quality has deteriorated, which slightly hinders viewing	3	Common
2 points	Obstructs viewing	2	Bad
1 points	Very serious hindrance to viewing	1	Very bad

## Appendix A

Let the equation of the ellipse be as follows:

(A1) $${Ax}^{2}{+Bxy+Cy}^{2}+Dx+Ey+F=0,$$

Let $W={\left[A,B,C,D,E,F\right]}^{T}$, and $X={\left[x^{2}{,xy,y}^{2},x,y,1\right]}^{T}$, then the optimization goal is as follows:

(A2) $$\min\|W^{T}X{\|}^{2}{=W}^{T}{\mathrm{XX}}^{T}W\;\mathrm{and}\;W^{T}\mathrm{HW}>0,$$

where

$$H=\left[\begin{array}{cc} \begin{array}{ccc} 0 & 0 & 2\\ 0 & -1 & 0\\ 2 & 0 & 0 \end{array} & \begin{array}{ccc} 0 & 0 & 0\\ 0 & 0 & 0\\ 0 & 0 & 0 \end{array}\\ \begin{array}{ccc} 0\; & 0 & \;0\\ 0\; & 0 & \;0\\ 0\; & 0 & \;0 \end{array} & \begin{array}{ccc} 0 & 0 & 0\\ 0 & 0 & 0\\ 0 & 0 & 0 \end{array} \end{array}\right]$$

$W^{T}\mathrm{HW}$> 0 is the constraint on the elliptic parameter; that is, ${4\mathrm{AC}-B}^{2}>0$. As $\|W^{T}X{\|}^{2}=0$, W can have a scaling factor; that is, W′=αW will also satisfy the condition, so let $W^{T}\mathrm{HW}=1$. Then, the optimization goal becomes

(A3) $$\min\|W^{T}X{\|}^{2}=W^{T}{\mathrm{XX}}^{T}W\;\mathrm{and}\;W^{T}\mathrm{HW}=1.$$

The Lagrange functions are constructed as follows:

(A4) $$L\left(W,\lambda \right){=W}^{T}{\mathrm{XX}}^{T}{W-\lambda (W}^{T}\mathrm{HW}-1).$$

Letting $\frac{\partial L}{\partial W}=0\;$,

(A5) $${\mathrm{XX}}^{T}W-\lambda \mathrm{HW}=0.$$

Letting ${S=\mathrm{XX}}^{T}$,

(A6) SW=λHW.

The eigenvalues and vectors ${(\lambda }_{i}{,u}_{i})$ of Equation (A6) are then solved. ${(\lambda }_{i}{,\mu u}_{i})$ is also the characteristic solution of Equation (A6), where μ is an arbitrary real number. Setting $\mu^{2}u_{i}^{T}Hu_{i}=1$ according to $W^{T}\mathrm{HW}=1$, gives

(A7) $$\mu =\sqrt{\frac{1}{u_{i}^{T}{Hu}_{i}}}=\sqrt{\frac{\lambda_{i}}{u_{i}^{T}{Su}_{i}}}.$$

By taking the eigenvector $u_{i}$ corresponding to $\lambda_{i}>0$, the coefficients of the fitted ellipse can be obtained. The ellipse’s center coordinates ${(X}_{c}{,Y}_{c})$ are

(A8) $$\{\begin{array}{c} X_{c}=\frac{BE-2CD}{{4\mathrm{AC}-B}^{2}}\\ Y_{c}=\frac{BD-2AE}{{4AC-B}^{2}} \end{array}\;,$$

and the long semi-axis *aa* and short semi-axis *bb* are

(A9) $$aa=\sqrt{\frac{{2(AX}_{c}^{2}{+CY}_{c}^{2}{+BX}_{c}Y_{c}-F)}{A+C-\sqrt{{\left(A-C\right)}^{2}{+B}^{2}}}},$$

(A10) $$.bb=\sqrt{\frac{{2(AX}_{c}^{2}{+CY}_{c}^{2}{+BX}_{c}Y_{c}-F)}{A+C+\sqrt{{\left(A-C\right)}^{2}{+B}^{2}}}}.$$

## References

[1] The Statistical Bulletin on the Development of the Transportation Industry in 2018. http://xxgk.mot.gov.cn/jigou/zhghs/201904/t20190412_3186720.html.

[2] Xu Z. (2009). Data Processing of Tunnel Engineering Deformation Monitoring. Master’s Thesis.

[3] Chen M. (2016). Research on Mass Data Processing and Application of Laser Scanning in Subway Shield Tunnel. Master’s Thesis.

[4] Wang T.T., Jaw J.J., Chang Y.H., Jeng F.S. (2009). Application and validation of profile–image method for measuring deformation of tunnel wall. Tunn. Undergr. Space Technol..

[5] Yun L. (2015). Study on Qualitative Evaluation of Map Quality Base on Vague Set. Build. Superv. Cost.

[6] Amberg Clearance GRP 5000. https://ambergtechnologies.com/solutions-services/amberg-rail/grp-system-fx/.

[7] Leica SiTrack: One. https://leica-geosystems.com/products/mobile-sensor-platforms/capture-platforms/sitrack_one.

[8] Cui H., Ren X., Mao Q., Wang W. (2019). Shield subway tunnel deformation detection based on mobile laser scanning. Autom. Constr..

[9] Nuttens T., Stal C., De Backer H., Schotte K., Van Bogaert P., De Wulf A. (2014). Methodology for the ovalization monitoring of newly built circular train tunnels based on laser scanning. Autom. Constr..

[10] Xie X., Lu X. (2017). Development of a 3D modeling algorithm for tunnel deformation monitoring based on terrestrial laser scanning. Undergr. Space.

[11] Xu X., Yang H., Neumann I. (2018). A feature extraction method for deformation analysis of large-scale composite structures based on TLS measurement. Compos. Struct..

[12] Kang Z., Zhang L., Tuo L., Wang B., Chen J. (2014). Continuous extraction of subway tunnel cross- sections based on terrestrial point clouds. Remote Sens..

[13] Du L., Zhong R., Sun H. (2018). Tunnel Cross Section Extraction and Deformation Analysis Based on Mobile Laser Scanning Technology. Surv. Mapp..

[14] Kang Z., Zlatanova L.S. (2012). Continuously Deformation Monitoring of Subway Tunnel Based on Terrestrial Point Clouds. Remote Sens. Spat. Inf. Sci..

[15] Lindenbergh R., Pfeifer N., Rabbani T. Accuracy analysis of the Leica HDS3000 and feasibility of tunnel deformation monitoring. Proceedings of the ISPRS Workshop on Laser Scanning, ITC.

[16] Delaloye D. (2012). Development of a New Methodology for Measuring Deformation in Tunnels and Shafts with Terrestrial Laser Scanning (LIDAR) Using Elliptical Fitting Algorithms. Master’s Thesis.

[17] Walon G., Delaloye D., Diederichs M.S. (2014). Development of an elliptical fitting algorithm to improve change detection capabilities with applications for deformation monitoring in circular tunnels and shafts. Tunn. Undergr. Space Technol..

[18] Li C., Lu X., Zhu N., Lu Y., Wu Y., Li G. (2015). Continuously Extracting Section and Deformation Analysis for Sunway Tunnel Based on LIDAR Points. Acta Geod. Cartogr. Sin..

[19] Jian L., Wan Y., Jiang M. (2012). Tunnel Deformation Monitoring Based on the Terrestrial Laser Scanning Technology. Geospat. Inf..

[20] Xie X., Lu X., Tian H., Ji Q., Li P. (2013). Development of a modeling method for monitoring tunnel deformation based on terrestrial 3D laser scanning. Chin. J. Rock Mech. Eng..

[21] Li J. (2015). Horizontal chord lenth testing of single circle shield tunnel based on point clouds. J. Zhejiang Univ. Water Resour. Electr. Power.

[22] Zhang L., Cheng X. (2018). Tunnel Deformation Analysis Based on Lidar Points. Chin. J. Lasers.

[23] Liu G., Xu Y., Bai W. (2016). A Method to Determine the Deformation of Tunnel Section. Surv. Mapp..

[24] Zhang F. (2018). Data processing and visualization analysis method of tunnel segment structure deformation based on laser scanning. Mod. Tunn. Technol..

[25] Wang L., Cheng X., Wang C. (2013). Study on the 3D laser scanning technology for tunnel inspection. Geotech. Investig. Surv..

[26] Lin Y. (2017). Research on Key Technology of Tunnel Point Cloud Data Processing and Visualization. Master’s Thesis.

[27] Stios S., Kontogianni V. (2009). Mean deformation tensor and mean deformation ellipse of an excavated tunnel section. Int. J. Rock Mech. Min. Sci..

[28] Fischler M.A., Bolles R.C. (1981). Random Sample Consensus: A Paradigm for Model Fitting with Applications to Image Analysis and Automated Cartography. Commun. ACM.

[29] Ministry of Housing and Urban-Rural Construction of the People’s Republic of China (2014). The Technical Specification for Urban Rail Transit Engineering Monitoring.

[30] Chen H., Li N., Wang X., Guo X. (2015). Introduction to Digital Media Technology.

[31] Yao F., Shao G., Takaue R., Tamaki A. (2003). Automatic Concrete Tunnel Inspection Robot System. Adv. Robot..

[32] Yu S.N., Jang J.H., Han C.S. (2007). Auto Inspction System Using a Mobile Robot for Detecting Concrete Cracks in a Tunnel. Autom. Constr..

[33] Puente I., Gonzalez-Jorge H., Martinez-Sanchez J., Arias P. (2014). Automatic Detection of Road Tunnel Luminaires Using a Mobile LiDAR System. Measurement.

